# AVPR1A Variant Associated with Preschoolers' Lower Altruistic Behavior

**DOI:** 10.1371/journal.pone.0025274

**Published:** 2011-09-28

**Authors:** Reut Avinun, Salomon Israel, Idan Shalev, Inga Gritsenko, Gary Bornstein, Richard P. Ebstein, Ariel Knafo

**Affiliations:** 1 Department of Neurobiology, The Hebrew University of Jerusalem, Jerusalem, Israel; 2 Department of Psychology, The Hebrew University of Jerusalem, Jerusalem, Israel; 3 S. Herzog Memorial Hospital, Jerusalem, Israel; 4 Center for the Study of Rationality and Interactive Decision Theory, Jerusalem, Israel; 5 Department of Psychology, National University of Singapore, Singapore, Singapore; University of Utah, United States of America

## Abstract

The genetic origins of altruism, defined here as a costly act aimed to benefit non-kin individuals, have not been examined in young children. However, previous findings concerning adults pointed at the arginine vasopressin receptor 1A (AVPR1A) gene as a possible candidate. AVPR1A has been associated with a range of behaviors including aggressive, affiliative and altruistic phenotypes, and recently a specific allele (327 bp) of one of its promoter region polymorphisms (RS3) has been singled out in particular. We modeled altruistic behavior in preschoolers using a laboratory-based economic paradigm, a modified dictator game (DG), and tested for association between DG allocations and the RS3 “target allele.” Using both population and family-based analyses we show a significant link between lower allocations and the RS3 “target allele,” associating it, for the first time, with a lower proclivity toward altruistic behavior in children. This finding helps further the understanding of the intricate mechanisms underlying early altruistic behavior.

## Introduction

Altruism, defined here as costly acts aimed at benefiting strangers, is a subgroup of prosocial behaviors (voluntary behaviors intended to benefit another; [1]). Prosocial behavior is an integral part of our everyday life as societal beings, helping us to establish and maintain interpersonal relationships. Human societies are often viewed as unique among the animal kingdom due to widespread exhibitions of other-oriented behaviors directed toward genetically-unrelated individuals [2]. The beneficial outcomes of prosocial behavior are evident in its positive association with academic achievement and in its negative association with aggression and externalizing problems ([1], [3], although negative consequences of extreme altruism are now acknowledged [4]). These advantageous effects of prosocial behavior on children's development stress the importance of understanding its genetic and environmental origins.

### Genetics of Altruism

Twin studies using various measures have shown that prosocial behavior exhibits moderate to considerable heritability [5]–[7]. One study for example [7] assessed adult twins' donations to a charity organization, and found a heritability effect of 31%. Nonetheless, only recently has the contribution of specific common genetic polymorphisms been shown with regard to individual differences in altruism using both questionnaire [8] and experimental measures [9], [10]. To our knowledge, even less research has been done on children. Only two previous studies have examined the association between a specific genetic polymorphism and preschoolers' prosocial behavior [11], [12], and their focus was not on altruistic behavior toward strangers. The studies using adult samples implicated both dopamine and vasopressin related genes, however only the results concerning dopaminergic genes have been investigated in children. Thus, in the current study we sought to further extend the limited data on the genetic underpinnings of altruism by examining the role of the arginine vasopressin receptor 1A (AVPR1A) gene in preschoolers.

### Altruism

Costly altruism presents some difficulty to evolutionary theorists, since the cost is not always recovered, thus resulting in what appears to be a reduction of fitness. Nevertheless, as noted by Adam Smith [13], altruism is widespread in human communities and under a wide range of conditions people are willing to deviate from strategies that maximize their material payoffs. A voluminous literature across multiple disciplines seeks to account theoretically for non-kin altruism (altruism toward genetically unrelated individuals), and different mechanisms have been proposed, including direct reciprocity, indirect reciprocity, network reciprocity, and group selection, each of which possibly accounting for altruism depending on pertinent situational constraints [14].

However, none of the theories seem equal to the challenge presented by the high rates of giving in the dictator game (DG; [9]). The DG is a one-shot game with two players: the “Dictator” and the “Recipient”. The dictator is given a fixed sum of money and has to decide how to split it between himself and the recipient. The recipient has to accept the decision of the dictator and does not have an active role in the game. Anonymity in the DG is meant to shield it from reputation-based decisions, and the fact that it is a one-shot game shields it from strategic considerations of reciprocity. Therefore the DG is a measure of ‘pure altruism’ in the economic sense, an altruistic behavior which is not dependent upon strategic considerations of reciprocity [15], and may thus be considered as representing untainted other-oriented motivations.

### The AVPR1A Gene

AVPR1A regulates the activity of arginine vasopressin (AVP). Across mammal species, AVP serves both as a peripheral hormone in regulating water balance as well as a hormone in the brain associated with social behaviors. The role of this gene has been reviewed in both animal [16] and human studies [17] of affiliative and altruistic behaviors.

The AVPR1A gene is characterized by three promoter region microsatellites ((GT)_25_, RS1 and RS3; [18]), which are simple sequence repeats (SSR) in a non-coding region. SSRs are repetitive DNA sequences in which a short base-pair motif is repeated several to many times in tandem (e.g. CACACACACA). They can affect processes such as translation and transcription [19] and can be highly polymorphic, thus considered to be a major source of inter-individual genetic and phenotypic variation [20]. Importantly, in the vole, the AVPR1A promoter region is part of a regulatory system modulating gene expression across brain areas responsible for social cognition [21]. Similarly, in humans the presence of long AVPR1A RS3 promoter region repeats predicts increased expression in post-mortem hippocampal specimens [9], increased amygdala activation [22] and increased prepulse inhibition in nonclinical subjects [23].

### The current study

A growing body of research associated the AVPR1A RS3 327 bp allele (or 334 bp allele, depending on the PCR amplification methods used in each study) with a range of social skills in both nonclinical and clinical groups [22], [24]–[26]. Our aim was to examine a possible association between the RS3 327 bp allele (‘target allele’) and altruistic behavior in preschoolers with a sample of 3.5 year-old twins. Altruistic behavior toward an unknown stranger was measured by employing the DG, which was modified to fit children by using sticker charts as the valued commodity. Since genetic data of families were at our disposal, we used both family and population based analyses.

## Methods

### Ethics Statement

Ethics approval for this study was obtained from the ethics committee at Herzog Hospital, Jerusalem, and by the Israeli Ministry of Health higher committee on human medical research. Mothers provided written informed consent before enrolling in the study.

### Participants

Participants were three and a half-year old twins, (*mean age* = 44.66 months, *SD* = 3.01). They were part of a larger sample of twins, participating in the Longitudinal Israeli Study of Twins, whose focus is on children's social development as influenced by genetic and environmental effects [27]. The details of Jewish families of twins born in 2004–2005 were obtained from the Israeli Ministry of the Interior after an IRB approval was obtained. When twins, living in Jerusalem and nearby surroundings, reached the age of 36 months, their families were contacted and invited to partake in the experiment. Ninety-eight twin pairs were included in the current study (34 monozygotic twin pairs and 64 dizygotic twin pairs). Out of these pairs we had phenotypic data on 158 twins, 77 males and 81 females (12 MZs and 26 DZs did not cooperate with the experimenter). Twins' zygosity was determined via DNA analyses. For the population-based analyses, one twin from each family was randomly picked in order to establish independence of cases. The family-based analysis included all DZ twins but only one MZ twin from each MZ pair, in order to avoid amplifying the genetic association results. Thus two samples of twins were created for further analyses, the first consisting of 98 twins (population-based) and the second of 136 twins (family-based).

### Experimental Procedure

The DG was modified to suit preschoolers, and attractive sticker charts replaced monetary units, as shown to be effective in previous studies [28], [29]. Upon arrival of the family members (usually the mother and the twins) to the lab, two female experimenters welcomed them and spent a few minutes getting acquainted with the twins [30]. Twins were next led separately to one of two identical rooms with one of the experimenters.

The DG was integrated into an interactive story which was read to each child. In the story, one of the characters finds an envelope and “asks” the reader to pass it on to the child listening to the story. After the envelope is given and opened to reveal 6 different charts of stickers, the child is asked the following – “all the stickers are yours, if you want you may give some of them to a child you do not know, who did not hear the story and did not get any stickers. Do you want to give stickers to a child you do not know and did not get any stickers?”. If the child did not seem to comprehend the question it was gently repeated up to three times in order to guarantee understanding. Experimenters were instructed to ask the child whether he or she wanted to donate any stickers, without pressuring them or showing an expectation of a specific behavior. The experimenter stressed that the sticker charts belonged to the child, and that he/she might give some of them only if he/she wished to do so. If the child expressed willingness to allocate some of his/hers stickers, then he/she was asked to put the allocated sticker charts in an envelope. The experimenter then took the envelope and put it away.

#### DNA Extraction and Genotyping

DNA was obtained from both twins and both parents when possible (34 fathers did not participate) and extracted with use of the MasterPure kit (Epicentre, Madison, Wisconsin, United States). Amplification of the RS3 AVPR1A microsatellites was achieved using the following primers [18], [31]: forward (fluorescent) 5′-CCT GTA GAG ATG TAA GTG CT-′3 and reverse 5′-TCT GGA AGA GAC TTA GAT GG-′3.

Each reaction mixture contained 0.5 µM primer and 20 ng of DNA. A ReddyMix master mix (Thermoprime plus DNA polymerase) was used (Abgene, Surrey, United Kingdom) at a magnesium concentration of 1.5–2.5 mM MgCl_2_. ReddyMix buffer consisted of 75 mM Tris-HCl (pH 8.8 at 25°C), 20 mM (NH_4_)_2_SO_4_, and 0.01% (v/v) Tween 20. The sample was initially heated at 95°C for 5 min followed by 30 cycles of 95°C (30 s), 55°C (30 s), and 72°C (40 s), and a final extension step of 72°C for 10 min. The PCR product was analyzed on an ABI 310 DNA analyzer (Applied Biosystems, Foster City, California, United States).

The observed length of the RS3 promoter region depends on the PCR primers employed, and the 327 bp allele in the current study corresponds to the 334 allele in other studies [22], [24]. In these studies and in the present investigation, this ‘target allele’ is the second most common RS3 variant (∼22%). Allele frequencies for RS3 are shown in Table 1. The frequencies are comparable to previous findings [9], [22].

**Table 1:** Frequencies of AVPR1A RS3 Promoter Region Repeat Alleles, in a Sample of 136 Twins and 162 Parents.

RS3 bp	Relative
	frequency
308	0.2
310	1.8
312	0.3
319	0.2
321	2.7
323	10.2
325	27.7
327	21.5
329	15.1
331	9.7
333	2.2
335	1.5
337	3.9
339	1.8
341	1.2

#### Statistical Analysis

Mendelian inheritance and Hardy-Weinberg equilibrium were verified with PEDSTATS version 0.6.12 [32] (http://www.sph.umich.edu/csg/abecasis/PedStats/). SPSS v. 16 (windows) was used for the population-based analysis. In a population-based design the frequencies of the alleles of interest are compared between populations of cases and controls, and a higher frequency in cases is taken as evidence that the allele is associated with the targeted phenotype [33].

In order to avoid the confounding effects of population stratification and further validate the findings, the transmission disequilibrium test (TDT) was used for family-based analysis. In the simplest family-based design which uses trios, affected offspring and two parents (in the current study we had genotypic and phenotypic data on both twins in 38 families, adding more information and thus more power to the design), the null hypothesis expects transmission according to Mendelian inheritance – all alleles having a 50% chance of being inherited to the next generation. A deviation from the expected allelic transmission indicates an association between the targeted alleles and the phenotype [34]. TDT analysis was performed with UNPHASED, version 3.0.13 (http://www.mrc-bsu.cam.ac.uk/personal/frank/software/unphased/; [35]).

## Results

There were no significant sex differences in allocations (t(df = 96) = 0.834, *p* = 0.4), therefore sex differences were not considered further.

The distribution of the number of sticker charts allocated is presented in Table 2. Since only a few children allocated more than one chart of stickers, they were grouped together. Allele frequencies for RS3 are shown in Table 1. The RS3 327 bp allele was the ‘target allele’ which we examined against all other alleles.

**Table 2:** Distributions of Allocated Sticker Charts in Two Subsamples.

Number of allocated	All DZ twins and a randomly picked		One randomly picked twin from each	
sticker charts	twin from each MZ pair		pair of twins	
	Frequency	Percentage	Frequency	Percentage
0	47	34.6	33	33.7
1	67	49.3	49	50.0
2	10	7.4	7	7.1
3	5	3.7	3	3.1
4	4	2.8	3	3.1
5	2	1.5	2	2.0
6	1	.7	1	1.0
Total	136	100.0	98	100.0

**Figure 1:**
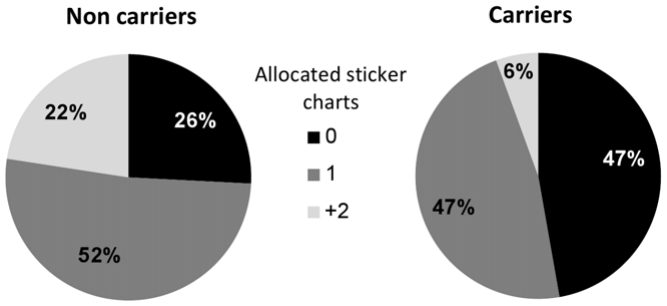
The effect of AVPR1A RS3 ‘target allele’ on allocations in the DG. Percentage of children according to the number of sticker charts allocated in the DG (0, 1 or 2 and more), and the presence/absence of the ‘target allele’. Compared with non carriers, carriers are much less likely to allocate more than 2 sticker charts, and more likely to allocate nothing.

Population-based analysis was used to examine the association between the RS3 ‘target allele’ and the number of sticker charts allocated. Three subjects were homozygous for the 327 bp allele, and were combined with the heterozygous, such that carriers of at least one copy of the ‘target allele’ were compared to non carriers (36 carriers vs. 62 non carriers). Kendall's tau-c was used due to the ordinal nature of the dependent variable. Results for the DG were significant (Kendall's tau-c = −.27, *p* = .004), with carriers of the 327 bp allele being 4 times less likely to donate more than one sticker chart (Fig. 1).

A family-based analysis was used in order to further examine the association between the ‘target allele’ and allocations. An important characteristic of family-based designs is their robustness against population substructure [34]. When comparing cases versus controls in a population-based design it is highly important that the groups would be a perfect match in every phenotype except for the one being tested (i.e. age, sex, ethnicity, disorders etc). This difficulty is avoided when using the family-based design. Indeed, it has been suggested that family and population-based designs should be combined in order to achieve greater power and overcome the challenges in elucidating the roles of specific genes in complex phenotypes [34].

As expected from the population based analysis, a significant association was found between the RS3 327 bp repeat, coded as ‘target allele’ vs. all others, and sticker chart allocations, χ^2^(1, *N* = 136) = 3.89, *p* = 0.04, indicating carriers as less likely to allocate sticker charts.

## Discussion

Young children's prosocial behavior is now increasingly understood as developing earlier than previously thought [36]. The current results, indicating that more than half of the children examined were willing to give one sticker chart or more (Table 2), add to the limited research pointing at altruistic behavior toward an anonymous stranger in as early an age as 3.5 [29], [37]. Gummerum, et al. [29] reported that moral emotions predicted preschoolers' allocations, and Blake and Rand [37] showed the effect of currency value. We add one more piece to the puzzle, by demonstrating the influence of preschoolers' genetic makeup, more specifically the presence or absence of the AVPR1A RS3 327 allele. This is the first study to show evidence for a measured association between a specific polymorphism and altruistic behavior in preschoolers.

The link between AVPR1A and social behavior in general, and altruism in particular, may be partially explained by the association found in adults between the length of RS3 and AVPR1A mRNA expression in the hippocampus [9]. The hippocampus is a part of the limbic system, and it was suggested to modulate information passing through to the ventral pallidum (VP; located within the mesolimbic dopamine reward pathway), while being modulated itself by dopaminergic receptors [38]. AVPR1A is also expressed in other regions associated with dopaminergic function and reward pathways (e.g. the striatum, the VP, and the medial prefrontal cortex) [39], thus a change in its expression pattern is likely to influence these systems as well. Decisions to donate to a charity are associated with activity in reward circuitry, which is compatible with the ‘warm glow’ effect, the rewarding feeling arising from the allocation of one's own property to another [40]. Therefore, a probable hypothesis is that the influence of AVPR1A on altruism is partly mediated by an interaction with reward mechanisms.

The new found link between the AVPR1A RS3 327 allele and children's lower allocations in the DG is in line with previous research that has also examined the RS3 ‘target allele’ effect individually and linked it with lower levels of social skills. Walum, et al. [26] measured scores for pair bonding (partner bonding, perceived marital problems, and marital status) using a questionnaire and found that carriers of the ‘target allele’ displayed significantly lower scores than subjects not carrying this allele. They examined 552 same-sex Swedish twin-pairs and their spouses/partners. Interestingly, the ‘target allele’ was also associated with marital status; the frequency of non-married men being higher among the ‘target allele’ homozygotes (32%) than among men who were non carriers (17%). Kim, et al. [24] reported an association between the ‘target allele’ and autism, which is characterized by deficits in social interaction and communication. This was supported by Meyer-Lindenberg, et al. [22] who associated the ‘target allele’ with higher amygdala activation. Thus, our finding adds to previous compelling research linking the ‘target allele’ to various aspects of social behavior.

The current finding partly replicates our previous finding in adults, which linked AVPR1A to altruism [9]. However the adult study, which used a short/long classification system based on the length of the RS3 repeat region, associated higher giving with variants classified as long, with alleles ranging from 327 to 343 bp. This seeming contradiction in the role played by the RS3 ‘target allele’ in altruistic behavior may be due to the age difference between the studied samples. Factors shown to influence prosocial behavior such as moral emotion [29], cognitive ability [41], and inequity aversion [42] tend to increase with age [29], [42], implying a change in the psychological factors underlying altruism. In addition, brain regions (e.g. the orbitofrontal cortex) associated with reward processing and decision making – both involved in altruistic behavior - develop and change with maturation [43]. Accordingly, it has been demonstrated that the genetic contributions to prosocial behavior vary by developmental stage [6]. Thus, it is possible that the specific effects of AVPR1A RS3 ‘target allele’ on altruism vary by age, suggesting a susceptibility to a yet undiscovered environmental or developmental influence.

### Strengths and Limitations

The main strength of the current study lies in the use of experimental data, specifically, an economic game which is applicable to various age groups and is being widely used in behavioral research. The use of families is also of value. It allowed the addition of a family-based analysis to the population-based analysis, and thus enabled the reliance on allelic transmission instead of solely on group comparisons. Another strength lies in the examination of preschoolers, which enabled the first finding of a main genetic effect on altruistic behavior in such an early age, that is not contingent upon possible reciprocation.

The main limitation of this study is the relatively small sample size. However, since the targeting of the AVPR1A gene was based on previous research linking it to social behavior in various samples [9], [24]–[26], [44], it is highly unlikely that the current results are based on chance, especially as two different analyses were used.

Similar to previous studies with children [28], [29] we have used stickers as monetary units. Stickers are a valued resource among preschoolers, and based on piloting we chose stickers that children found as attractive. However, unlike the DG protocol used in the current research, experimenters in the mentioned studies either covered their eyes or turned around while the child decided how much to donate. The presence of the experimenter (with her eyes opened) in the room did not seem to affect the frequency of giving, which was similar in our study (66%) to that reported in these studies for 3 and 4-year olds, (60%, χ2(df = 1) = 1.60, *p* = 0.20). Nonetheless, future research should investigate the association between the RS3 ‘target allele’ and preschoolers' altruism in a more private setting.

### Conclusions

An accumulating body of research shows AVP's role in social and affiliative behaviors across a diversity of species [16]. The current investigation extends the evidence for the role of AVPR1A in humans by linking it to social behavior in early childhood. Further research is needed in order to unravel the mechanisms behind AVP's involvement in human social behavior and clarify the role of this neuropeptide's interaction with the dopaminergic system. Additionally, longitudinal studies, incorporating molecular genetics and epigenetics, would facilitate the understanding of the genetic and environmental mechanisms underlying altruism across different developmental stages.
